# Binding-Induced Diversity of a Human Telomeric G-Quadruplex Stability Phase Space

**DOI:** 10.3390/ph15091150

**Published:** 2022-09-15

**Authors:** Domen Oblak, San Hadži, Črtomir Podlipnik, Jurij Lah

**Affiliations:** Faculty of Chemistry and Chemical Technology, University of Ljubljana, Večna pot 113, SI-1000 Ljubljana, Slovenia

**Keywords:** G-quadruplex, human telomere repeat, G-quadruplex stability, G-quadruplex ligand binding, G-quadruplex structure, thermodynamics, DSC

## Abstract

The structural polymorphism of G-quadruplex nucleic acids is an important factor in their recognition by proteins and small-molecule ligands. However, it is not clear why the binding of several ligands alters G-quadruplex topology. We addressed this question by following the (un)folding and binding of the human telomeric fragment 5′-(GGGTTA)_3_GGGT-3′ (22GT) by calorimetry (DSC, ITC) and spectroscopy (CD). A thermodynamic analysis of the obtained data led to a detailed description of the topological phase space of stability (phase diagram) of 22GT and shows how it changes in the presence of a specific bisquinolinium ligand (360A). Various 1:1 and 2:1 ligand–quadruplex complexes were observed. With increasing temperature, the 1:1 complexes transformed into 2:1 complexes, which is attributed to the preferential binding of the ligand to the folding intermediates. Overall, the dissection of the thermodynamic parameters in combination with molecular modelling clarified the driving forces of the topological quadruplex transformations in a wide range of ligand concentrations and temperatures.

## 1. Introduction

We normally associate the DNA molecule with the famous Watson–Crick double-helix structure [1]. However, certain G-rich sequences can form quite different non-canonical DNA structures, called G-quadruplexes (G4), which consist of stacked guanine tetrads stabilized by cation coordination [2,3,4]. Although G4 were first identified at chromosome ends, most G-rich DNA sequences occur in the context of double-stranded DNA. The complementary C-rich strand may form the i-motif, a noncanonical structure in which two parallel duplexes are intercalated in an antiparallel manner and are stabilized by three hydrogen bonds between cytosines [5,6,7,8,9,10,11]. The existence of G4 and i-motif structures has been confirmed *in vivo* by detecting them in cells with specific antibodies [12,13]. In addition, a novel noncanonical DNA structure consisting of G-rich strands with the AGCGA motif was recently identified [14,15,16].

Among the noncanonical structures, G4 have attracted particular attention because of their biological significance as potential regulators of transcription of cancer cell DNA sequences in gene promoter regions of oncogenes and in telomeric regions [17,18,19,20]. The formation of G4 and thus their ability to regulate transcription may be modulated by the specific binding of small-molecule ligands that are often considered as potential therapeutics [5,21,22,23,24,25,26,27]. The characterization of the binding affinity and selectivity of ligands for G4 is therefore of paramount interest.

**Figure 1:**
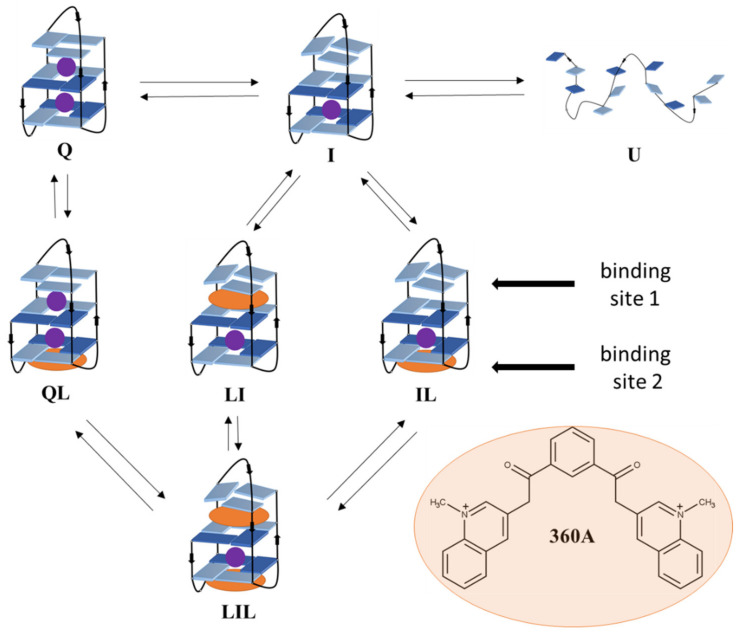
The simplest model mechanism that successfully describes temperature- and ligand (L = 360A) binding-induced structural conversions of 22GT. It includes folded species (Q, QL) with two specifically bound potassium ions, intermediate species (I, LI, IL, LIL) with one specifically bound potassium ion and an unfolded form (U). All the presented species are considered to be in equilibrium.

G4 are structurally heterogeneous, as demonstrated for a number of sequences in the absence and presence of ligands [28]. This structural heterogeneity is best characterized for human telomere DNA sequences. For example, X-ray crystallography showed that the fragment 22AG (=Tel22) adopts a parallel structure in the presence of K^+^ ions [29], whereas NMR and biophysical techniques indicated that 22AG exists in K^+^ solutions at room temperature as a mixture of two energetically similar (3+1) hybrid-type G-quadruplex structures known as hybrid-1 and hybrid-2 conformations [30,31,32]. On the other hand, it has been demonstrated by NMR that 22AG adopts an antiparallel structure in solutions containing Na^+^ ions [33]. Moreover, it has been shown that the folding of 22AG proceeds through several stable intermediate states [34,35,36]. After the binding of a ligand, the conformational phase space of 22AG may become even more complex, as several popular ligands are capable of inducing major changes of the quadruplex topology [37]. The bisquinolinium ligands 360A and Phen-DC3 have been shown to have a high affinity for G4, most likely due to strong stacking interactions with G-quartets [38]. Upon binding to the 22AG fragment, they select the antiparallel G4 structure with two G-quartets and only one specifically bound potassium ion [37,39,40]. The G4 topology changes at low temperatures and relatively high concentration of potassium ions (>10 mM) are per se unfavorable, but may be achieved when coupled to ligand binding, as observed for Phen-DC3 or 360A binding to 22AG [37,39,40]. These binding-coupled topology changes are expected to be less pronounced when the temperature is increased to the physiological range where slipped (e.g., two-quartet) folding intermediates become significantly populated. In this light, the thermodynamic signatures of binding of 360A to the human telomeric fragment 22GT which has high propensity to form the two-quartet antiparallel quadruplex is expected to be accompanied by rather small conformational contributions. To test this hypothesis, we investigated the (un)folding of the 22GT fragment and the binding of 360A to this sequence (see Figure 1) using differential scanning calorimetry (DSC), isothermal titration calorimetry (ITC) and circular dichroism (CD) spectroscopy. A global analysis of the measured data led to the thermodynamic parameters of 22GT folding and binding, which were decomposed into specific driving forces and interpreted using molecular modeling. The thermodynamic driving forces suggested the reason and the extent to which ligand binding to the 22GT fragment was accompanied by conformational changes in given solution conditions. Overall, our thermodynamic analysis explains how and to what extend does ligand binding alter the conformational stability phase space of human telomere quadruplexes.

## 2. Results and Discussion

### 2.1. Three Significant Species Are Populated during Melting in the Absence of Ligand

**Figure 2:**
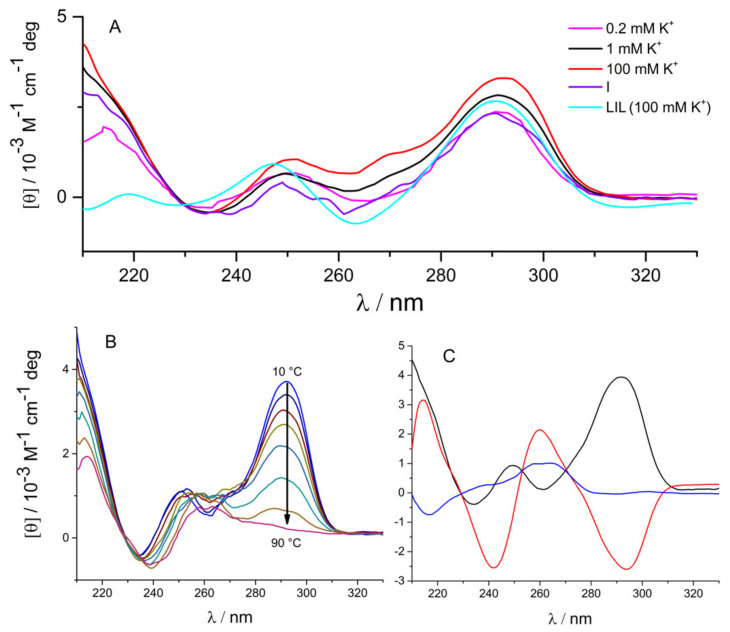
CD spectra of 22GT. (**A**) Spectra measured at 20 °C and different concentrations of potassium ions. The spectrum at [K^+^] = 0.2 mM was measured in a 100 mM TMAA solution [43]. The I spectrum corresponding to the model-predicted folding intermediate was estimated as described in the text. LIL spectrum of 22GT saturated with the ligand 360A; (**B**) Spectra measured at different temperatures at [K^+^] = 10 mM. (**C**) SVD analysis of the thermal unfolding spectra presented in panel B result in three dominant eigenvalues presented as spectra in black, red and blue.

The CD spectra of 22GT measured at 20 °C in solutions containing K^+^ ions are characterized by two maxima at 290 nm and 245 nm and two minima at 265 nm and 235 nm (Figure 2A). These characteristics are consistent with the formation of G4 structures with an antiparallel strand orientation [41]. The CD spectrum measured in solution with 100 mM concentration of K^+^ ions ([K^+^] = 100 mM) showing an additional shoulder at 270 nm corresponds to the hybrid-3 structure in which a triplet of guanines is stacked on the two-quartet quadruplex core [42]. This structure can bind specifically two K^+^ ions [43]. The CD spectrum measured at [K^+^] = 0.2 mM, according to mass spectrometry analysis, corresponds to the two-quartet antiparallel structure with only one specifically bound K^+^ ion [43]. Figure 2A shows that the CD spectrum measured in solution with [K^+^] = 1 mM differs from those collected at [K^+^] = 100 mM and [K^+^] = 0.2 mM, which may be due to the coexistence of the hybrid-3 structure (denoted as Q, see Figure 1) and the two-quartet structure that accommodates only one specifically bound K^+^ ion (denoted as I, see Figure 1) [37,42,43]. Indeed, the spectrum is successfully described as a linear combination of the spectra corresponding to the structures Q ([K^+^] = 100 mM) and I ([K^+^] = 0.2 mM). Accordingly, in the measured solution (20 °C, pH 7, [K^+^] = 1 mM), about 60% of the 22GT fragment exists in the Q form and 40% in the I form.

To examine the number of significantly populated species upon 22GT fragment unfolding in the model-independent way, we performed singular-value decomposition (SVD) [44] on data sets that included spectra measured at different temperatures (Figure 2B). The SVD analysis resulted in three dominant singular values (Figure 2C), suggesting that three species contributed significantly to the CD spectrum of the 22GT fragment in the measured temperature range.

**Figure 3:**
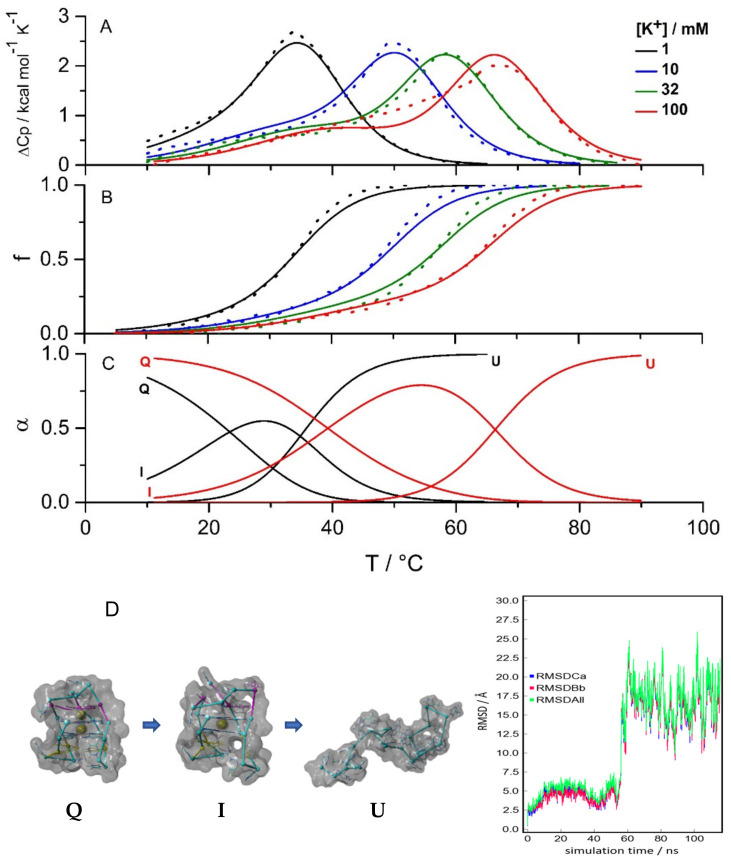
22GT unfolding monitored by DSC (**A**) and CD spectroscopy (**B**; melting curves measured at 290 nm normalized according to Equation (1)) at different [K^+^]. The globally fitted Q ↔ I ↔ U model functions (lines) show good agreement with the experimental data (symbols). (**C**) Model-predicted fractions of species at [K^+^] = 1mM (black curves) and [K^+^] = 100 mM (red curves); (**D**) Structural models of Q, I and U obtained from a molecular dynamic simulation of 22GT. RMSD of 22GT from the starting structure Q (corresponds to structure with PDB entry: 2kf8) during the MD simulation at elevated temperature.

In addition, unfolding was monitored at different [K^+^] by DSC and via temperature dependence of the CD signal at 290 nm (Figure 3). Consecutive DSC and CD melting scans agreed within experimental error margins. Thus (un)folding could be treated as a reversible process in our analysis. The DSC and CD spectroscopy melting curves measured at [K^+^] ≥ 10 mM were clearly not “monophasic” and could not be described by a two-state model. Based on the analysis of the CD spectra measured at different [K^+^] (Figure 2A) and on the SVD analysis (Figure 2C), we assumed that the observed unfolding involves three species: Q, I and the unfolded form U. The successful global fitting of the equilibrium Q ↔ I ↔ U model to the DSC and CD melting data fully confirms our assumption (see refs. [35,36,40,45] for details on the applied global analysis of the data). The model-predicted fractions of Q and I at [K^+^] = 1 mM and 20 °C (Figure 3C) agree well with the estimate based on the analysis of the CD spectra described above. It can be seen in Figure 3C that Q and I at [K^+^] = 1 mM disappeared concertedly when increasing the temperature, which is in accordance with the corresponding mass spectrometry analysis by the Gabelica group [46]. In the measured temperature range in the absence of ligand, the 22GT and 22AG fragments occupy similar structures Q (two specifically bound K^+^ ions), I (two-quartet, one specifically bound K^+^ ion) and U (unfolded, no specifically bound K^+^ ions). In addition, the CD spectrum of intermediate I was estimated from the spectra measured at different temperatures (Figure 2B). The measured spectra were described as a linear combination of the spectra of Q ([K^+^] = 10 mM, low temperature), I (unknown) and U ([K^+^] = 10 mM, high temperature) weighted by the corresponding model-predicted species fractions (Figure 3). The estimated spectrum of I shown in Figure 2A is very similar to that measured at [K^+^] = 0.2 mM, suggesting that I represents the two-quartet quadruplex structure with antiparallel strand orientation. The obtained thermodynamic parameters (Table 1) suggest that the 22GT folding is similarly to one of the 22AG fragment accompanied by an extensive enthalpy–entropy compensation and a negative heat capacity change [47]. However, the enthalpy change accompanying the first stage of folding (U → I) is significantly more negative (favorable) than that accompanying the second (I → Q) stage. In the case of the 22AG fragment, the first stage of folding is less enthalpically favorable than for 22GT [36]. The Gibbs free energies of 22GT transitions (Table 1) suggested a higher tendency to form I from U and a lower tendency to form Q from I than in the case of 22AG [36]. In other words, the 22GT fragment could form a two-quartet structure that is more stable than the corresponding two-quartet structure formed by the 22AG fragment under the same solution conditions.

To obtain additional information on 22GT (un)folding, we performed molecular modelling and molecular dynamics simulations of this fragment. We assumed that the folded (Q) form corresponded to the two-quartet hybrid-3 NMR structure containing two non-equivalent potassium ion binding sites—the high-affinity site between the two G-quartets, and the low-affinity site located between the G-quartet and the triplet of guanines [42]. Potassium ions were added to the corresponding binding sites, and simulation was performed in a 100 mM KCl solution at 25 °C. The simulation results confirmed the stability of the folded Q structure in the chosen conditions. Next, a molecular dynamics simulation was performed at elevated temperature to monitor the conformational changes accompanying the unfolding of the starting structure (Q). The RMSD plot (Figure 3D) and deviations in the number of intramolecular hydrogen bonds suggested the presence of an intermediate in the timeframe between 40 ns and 55 ns. The intermediate average structure (I) presented in Figure 3D was calculated from the simulation snapshots in this timeframe. The proposed I structure contains only two G-tetrades, with one K^+^ ion coordinated between them. The low-affinity K^+^ binding site is empty, and there is no additional stacking of the triplet of guanines on the G-quartet, which is in accordance with the absence of a maximum at 270 nm and the presence of a minimum at 260 nm in the presented CD spectra (Figure 2A). The unfolded structure (U), with an occasional stacking of bases, was observed in simulations after 60 ns.

### 2.2. Driving Forces of Ligand Binding

**Figure 4:**
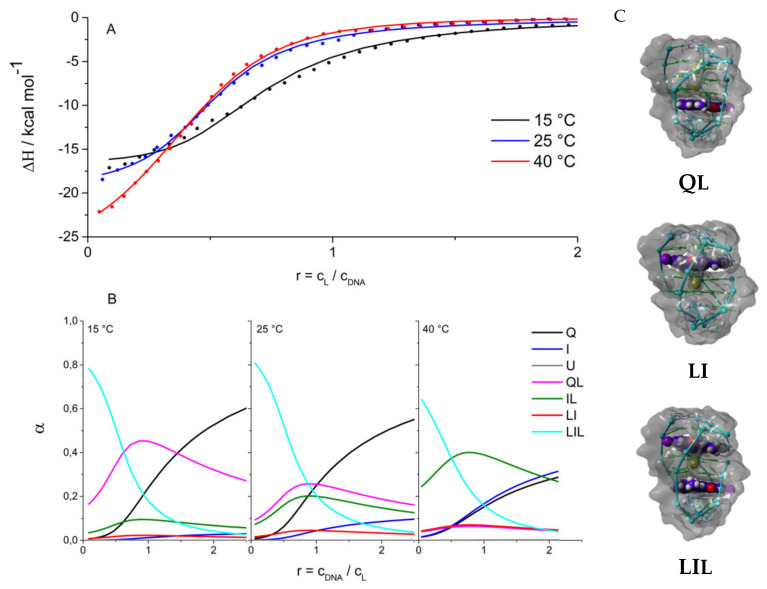
Ligand (306A) binding to 22GT: (**A**) ITC curves (dots) and global fits (lines) of the model function based on the mechanism presented in Figure 1. (**B**) Species present at different temperatures and ligand concentrations calculated using best fit values of the thermodynamic parameters (Table 2). (**C**) Structural models obtained by docking of the ligand to 22GT structures obtained by molecular dynamics simulations (Figure 3).

Due to the low solubility of the 360A ligand in buffer solutions, its binding was investigated using calorimetric titrations, in which the 22GT fragment solution was titrated into the 360A solution at temperatures between 15 °C and 40 °C and at [K^+^] = 100 mM (Figure 4). In these conditions, Q is the dominant species, and the U state is not significantly populated (Figure 3C). It was not possible to satisfactorily describe these isotherms with the model that assumes two non-equivalent independent binding sites or with the model assuming the sequential binding of two ligand molecules to Q. Further analysis of different binding models suggested that the simplest model able to describe the data is the one presented in Figure 1. It assumes the coexistence and interconversion between Q, I, QL, LI, IL and LIL species. The structural features of ligand-free 22GT fragment reported above (Figure 1 and Figure 3C) suggest that it can accommodate one 360A molecule on the “upper” end (binding site 1) and one on the “bottom” end (binding site 2). Mind that in our notation, the position of the L index refers to complexes with different binding sites, i.e., in LI, the ligand is bound to the upper site, while in IL, it is bound to the bottom site (as in QL). Previous binding studies with biquinolinium ligands suggested that site 1 can be occupied by the ligand only after the specifically coordinated potassium is removed from the binding site. Therefore, the binding of one 360A molecule to site 1 can be considered as LI complex formation. The LQ complex, with two specifically bound potassium ions has not been observed experimentally [37] and was therefore not considered in our model. Due to similar structural characteristics of the bottom ends of Q and I, site 2 was considered to be equivalent in both Q and I forms. Accordingly, the Q + L → QL, I + L → IL and LI + L → LIL events (binding to site 2) were assumed to have the same thermodynamic characteristics. The thermodynamic parameters of binding reported in Table 2 indicate that the binding of the ligand to site 1 is more favorable than that to site 2. The binding to both sites is enthalpy-driven. The corresponding enthalpy, entropy and heat capacity of binding are more negative for binding to site 2. The estimated binding affinities of 360A for 22GT between 10^7^ M^–1^ and 10^6^ M^–1^ are in the range observed for the binding of bisquinolinium ligands to various G4-forming sequences under similar solution conditions [37,38,39,40,46,48,49,50,51].

Next, we attempted to analyze the apparent standard Gibbs free energy of binding, ∆*G* (Table 2), taking into account various more fundamental driving forces. We considered ∆*G* as the sum of three main contributions: ∆*G* = ∆*G*_hyd_ + ∆*G*_int_ + ∆*G*_other_. The ∆*G*_hyd_ contribution refers to the (de)solvation/effects. At 25 °C, it is considered to reflect mainly the entropy of dehydration of hydrophobic groups upon ligand binding and may be estimated as ∆*G*_hyd_ = 80 (±10) K∆*C_p_* [52,53]. The Δ*G*_int_ contribution is ascribed to the specific inter- and intra-molecular interactions (π-π stacking, H-bonds, electrostatic). It can be considered mainly as an enthalpic contribution [39] and as such approximated by the measured enthalpy change Δ*G*_int_ ≈ Δ*H*. The ∆*G*_other_ is considered as the sum of two main contributions, i.e., the unfavorable entropy contribution due to changes in rotational and translational degrees of freedom upon ligand binding and the entropy contribution that may be ascribed predominantly to the conformational changes of the 22GT fragment accompanying 360A binding. The calculation of ∆*G*, ∆*G*_hyd_ and ∆*G*_int_ as described above enabled the estimation of ∆*G*_other_ = ∆*G* − ∆*G*_hyd_ − ∆*G*_int_.

The decomposition of the binding energetics reveals some general features of the binding of 360A to the 22GT fragment (Table 2). The binding of the ligand to site 2 is accompanied by a negative change in enthalpy and heat capacity, suggesting that the specific interactions occurring at the binding interface and the removal of water from this interface play a major role in the formation of the QL and IL complexes. This is consistent with the observation that the corresponding ∆*G*_hyd_ and ∆*G*_int_ contributions overcompensate the unfavorable rotational, translational and conformational contributions: (∆*G*_hyd_ + Δ*G*_int_) < −∆*G*_other_. Similar features were observed for 360A binding to the 22AG fragment [36]. The decomposition further suggests that the dominant forces of binding of 360A to site 1 (LI complex formation) are specific intra- and intermolecular interactions. The contribution of dehydration is rather small. It is reasonable to assume that binding to the two sites is accompanied by a similar loss of translational and rotational freedom of the ligand and DNA. With this in mind, the difference between the ∆*G*_other_ contributions can be attributed mainly to different contributions of the conformational changes associated with ligand binding to site 1 and site 2. It appears that binding to site 2, similarly to that to the 22AG fragment, is accompanied by DNA conformational changes that are significantly higher than those observed for binding to site 1. This is in line with the structural model (Figure 4C) developed by molecular modelling, which suggests that only ligand binding to site 2 requires a significant rearrangement of bases. The binding site 1 is accessible only when 22GT is in the form of the intermediate I. This site is more exposed than site 2; therefore, the binding is accompanied by the removal of a smaller amount of water molecules from the ligand–22GT binding interface, compared to site 2. The binding to site 2 is accompanied by an extensive burial of 360A in the G4 structure, which results in a more negative binding heat capacity than the one observed for site 1. Taken together, the binding of 360A to binding site 1 with high affinity is responsible for the formation of the ligand complexes with the two-quartet quadruplex having an antiparallel strand orientation (intermediate I). This is consistent with the observed characteristic CD spectra of the complex (LIL in Figure 2A) and with binding studies of bisquinolinium ligands by the Gabelica group [37].

### 2.3. Ligand Binding Alters the Stability Phase Space of 22GT 

**Figure 5:**
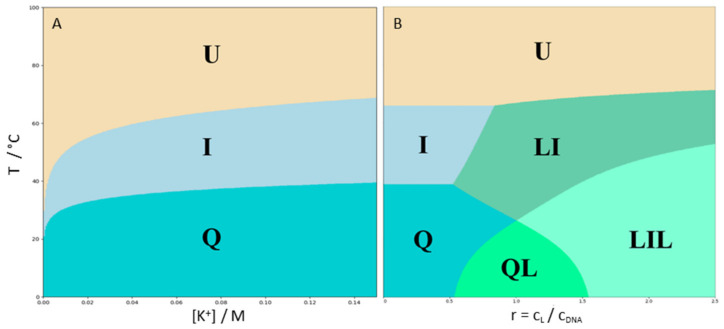
Phase diagrams presenting the most populated 22GT species (phases) at given T, [K^+^] and ligand/DNA molar ratios, r. C_L_ and C_DNA_ are the total molar concentrations of the ligand (360A) and DNA (22GT), respectively. (**A**) r = 0, C_DNA_ = 10 μM; (**B**) [K^+^] = 100 mM, C_DNA_ = 10 μM.

The obtained set of parameters (Table 1 and Table 2) was used for the calculation of the 22GT species fractions in the solution as a function of r, T and [K^+^], in a similar way as presented in refs. [36,54]. The most populated species at given r, T and [K^+^] was considered as a pseudophase. The corresponding 2D (T versus [K^+^], T versus r) phase diagrams were constructed by assigning areas in the phase space to the most populated species in that area, i.e., the pseudophase (Figure 5). Phase curves (borders) and triple points represented states in which two (curves) or three (triple points) of the most populated species (pseudophases) are equally populated. These diagrams indicated the possible macroscopic pathways of the 22GT fragment binding and structural conversions.

Figure 5a shows that the 22GT thermal unfolding followed the Q → I → U pathway at any [K^+^] and that at low [K^+^], the intermediate I is significantly populated at physiological temperatures. Recent thermal unfolding studies on similar human telomere DNA sequences [14,15,36] suggested that the phase populated by intermediates (phase I) in the presence of K^+^ may consist of several intermediate species, not only a single one, as described by our model mechanism (Figure 1). Despite this difference, which can be attributed to the different sensitivities of the methods used to monitor the unfolding pathway, the predicted temperature range at which phase I was stable matches the previous estimates for several human telomere variants [15,36]. The presented phase diagram shows that 22GT adopts an ensemble of structures that could be perturbed by ligand binding. How this happens is shown on the T-versus-r diagram (Figure 5B). At physiological temperatures and [K^+^] = 100 mM, an increase in the ligand concentration induces the formation of the LI complex which becomes the most populated species at r ≈ 1. Further increasing the ligand concentration induces the formation of the LIL complex that becomes the dominant solution species at r > 1.5. Such a behavior would be expected for the ligand binding predominantly to the I form, despite the Q form being the most populated species in these solution conditions in the absence of the ligand (Figure 5A). This is consistent with the preferential binding of 360A to the two-quartet quadruplex with one specifically bound potassium ion (binding site 1). At low temperatures in the r range 1 ≤ r ≤ 1.5, the QL species was dominant. Interestingly, increasing the temperature at these r values induces the formation of the LIL complex containing two bound ligand molecules. How can increasing the temperature induce the binding of an additional ligand molecule? The reason for this is twofold. Since the reaction Q → I is endothermic, the fraction of I increases with the increase in the temperature. In addition, the binding to I is accompanied by Δ*H*, which was less negative than that for the binding to Q. Therefore, at higher temperatures, the difference in the binding affinity of the ligand to I compared to Q increases. This is the reason for the dissociation of some amount of ligand from the QL complexes and a conformational rearrangement leading to a predominant type of complex with two ligand molecules bound to the intermediate I.

## 3. Materials and Methods

### 3.1. Sample Preparation

The HPLC-pure oligonucleotide 5′-GGGTTAGGGTTAGGGTTAGGGT-3′ (22GT) was purchased from Midland Co., Midland, TX, USA. The buffer solutions used in our experiments consisted of 1 mM cacodylic acid, 0.02 mM EDTA and various amounts of K^+^ ions. KOH was added to cacodylic acid and EDTA to reach pH 6.9. Then, KCl was added to obtain the desired concentration of K^+^ ions. 22GT was first dissolved in water and extensively dialyzed against the buffer using a Float-A-Lyser dialysis tube (Spectrum Laboratories, Piscataway, NJ, USA, molecular weight cutoff 500–1000 Da). The dialyzed solution was first heated to 95 °C in an external thermostat to ensure that all DNA was in the unfolded state and then cooled to 25 °C at a cooling rate of 0.05 °C/min to allow the DNA to fold into the appropriate quadruplex structures. DNA concentration was determined before each experiment at 25 °C using the Cary 100 BIO UV/Visible Spectrophotometer (Varian, Cary, NC, USA) equipped with a thermoelectric temperature controller. The molecular extinction coefficient was determined using a nearest neighbor calculation for single-stranded DNA at 25 °C and 260 nm, resulting in an extinction coefficient of 223,500 M^−1^ cm^−1^ [55]. Absorbance at 260 nm of the denatured oligonucleotide obtained by the UV melting experiment, extrapolated to 25 °C, was used in the determination of G4 concentration together with the corresponding extinction coefficient. The bisquinolinium ligand 360A was a generous gift from prof. J. Plavec. It is poorly soluble in aqueous solution. It was first dissolved in DMSO and then transferred to a buffer solution. The lowest possible content of DMSO in the buffer solution, which was sufficient for the dissolution of the ligand at a given concentration, was 3%. Therefore, all amounts of ligand and 22GT were dissolved in the buffer solution containing 3% DMSO for the titration experiments. The ligand concentration was determined by measuring the absorbance at 25 °C and using the extinction coefficient of 5980 M^−1^ cm^−1^ at 370 nm.

### 3.2. Circular Dichroism Spectroscopy

The CD spectra were measured using the CD JASCO J1500 spectrophotometer. All CD spectra of the folded 22GT were measured in a 0.5 cm cuvette at 20 °C in the wavelength range between 210 and 330 nm. The oligonucleotide concentration was about 10 µM in all samples. The scanning rate was set to 20 nm min^−1^, and the signal averaging time was 4 s. The thermal unfolding of 22GT in different buffer solutions was monitored at the spectral maximum of 290 nm in the temperature range between 5 °C and 95 °C with a heating rate of 1 °C min^−1^. The measured ellipticity at a given wavelength corrected for the corresponding buffer contribution was normalized to 1 M DNA and optical pathlength of 1 cm. The obtained $\left[\theta \right]$ can be expressed as a linear combination of species fractions ($\alpha_{Q},\;\alpha_{I},\;\;\alpha_{U}$); by taking into account that $\alpha_{Q}=1-\alpha_{I}-\alpha_{U}$, one obtains the expression for the normalized CD signal [35]:

(1) $$f=\frac{\left[\theta \right]-{\left[\theta \right]}_{Q}}{{\left[\theta \right]}_{U}-{\left[\theta \right]}_{Q}}=\alpha_{I}f_{I}+\alpha_{U}$$

in which ${\left[\theta \right]}_{Q}$ and ${\left[\theta \right]}_{U}$ are obtained as pre-transitional (low *T*) and post-transitional (high *T*) baselines linearly extrapolated over the whole measured *T* range, and $f_{I}$ is considered as a temperature-independent normalization coefficient which was treated in the model analysis as an adjustable parameter [35].

### 3.3. Differential Scanning Calorimetry

Differential scanning calorimetry experiments were performed using the Nano DSC instrument (TA instruments, New Castle, DE, USA) at a DNA concentration of 100 μM in the temperature range between 0.5 °C and 95 °C with a heating and cooling rate of 1 °C min^−1^. The corresponding baseline thermograms (buffer–buffer) were subtracted from the sample scans and normalized to 1 mol of DNA to obtain the partial molar heat capacity of DNA as a function of temperature. In the DSC experiments, the measured quantity was the partial molar heat capacity of DNA, ${\bar{C}}_{P,\mathrm{DNA}}$ which is more conveniently expressed in terms of excess heat capacity, $\Delta C_{P}={\bar{C}}_{P,\mathrm{DNA}}-{\bar{C}}_{P,\mathrm{int}}$ where ${\bar{C}}_{P,\mathrm{int}}$ represents an intrinsic heat capacity of 22GT formally defined as ${\bar{C}}_{P,\mathrm{int}}=\alpha_{Q}{\bar{C}}_{P,Q}+\alpha_{I}{\bar{C}}_{P,I}+\alpha_{U}{\bar{C}}_{P,U}$. In the measured temperature interval, ${\bar{C}}_{P,\mathrm{int}}$ was approximated by the second-order polynomial on *T*, fitted to the low-temperature (folded Q form) and high-temperature (unfolded U form) parts of the experimental ${\bar{C}}_{P,\mathrm{DNA}}$. The obtained ${\bar{C}}_{P,\mathrm{int}}$ was subtracted from ${\bar{C}}_{P,\mathrm{DNA}}$ to obtain the excess $\Delta C_{P}$ (see SI of ref. [35] for details). The model analysis of the CD melting curves suggests that in the DSC measuring conditions, the pure Q form ($\alpha_{Q}=1$) was only present, at low temperatures, at the highest applied K^+^ ion concentration (100 mM). Therefore, the low-temperature part of ${\bar{C}}_{P,\mathrm{int}}$ was for all presented curves obtained from the thermogram measured at 100 mM K^+^ ion concentration. The excess heat capacity, $\Delta C_{P}$, could also be calculated for the suggested model mechanism of transitions (Q ↔ I ↔ U) and fitted to the experimental thermograms as we explained in references [35,36,40].

### 3.4. Isothermal Titration Calorimetry

Isothermal titration calorimetry experiments were performed in the temperature range between 15 °C and 40 °C by titrating a solution of 22GT (c_DNA_ = 70 μM) into a ligand (360A) solution (c_L_ = 10 μM)) using a VP-ITC isothermal titration calorimeter from Microcal (Northampton, MA, USA). The area under the peak after each injection of DNA solution was determined by automatic integration of the raw signal using the NITPIC software. The corresponding heat of dilution was subtracted, and the obtained heat effect was expressed per mole of DNA added per injection to calculate the enthalpy of interaction, ΔH. The enthalpy of interaction was also calculated for the suggested model mechanism of transitions (Figure 1) and fitted to the experimental data, as we explained in ref. [39].

### 3.5. Molecular Modelling

Yasara structure was used for the molecular docking and molecular dynamics simulations of the DNA fragment and its various complexes with the ligand [56]. For DNA modeling, we used the structure of 22GT in potassium solution solved by Lim et al. (PDB entry: 2KF8) [42]. First, the two potassium ions were inserted into the structure, one between the guanine tetrads, and the other between the guanine tetrad and the guanine triplet, in accordance with the mass spectroscopy measurements [37]. Next, the structure was energy-minimized using the AMBER 14 force field with the recently developed parmbsc0 parameters using the explicit water model TIP3P [57,58]. In the structure minimization procedure, K^+^ ions were used to neutralize the system, and an appropriate number of K^+^ and Cl^−^ ions were added to mimic the conditions in a solution containing 100 mM K^+^ ions. In the next step, coronene was used as a “pore-forming ligand” [39]. It was placed either in the middle between the guanine tetrad and the AAG triplet or in the place of the potassium ion between the guanine tetrad and the guanine triplet. Restrained minimization of the complex followed, which included the inserted coronene with a fixed conformation. Next, coronene was removed from the complex, and the resulting open conformation of 22GT was used as the receptor structure for the molecular docking experiments. For molecular docking, we used the AutoDock Vina program (implemented in Yasara structure) to obtain the structural models of the G4–ligand complexes [59]. We performed 50 independent docking experiments with a flexible ligand and a rigid receptor. The pose with the best score was energy-minimized using the same parameters as described above.

The molecular dynamics simulations were performed for the different predicted G4–ligand complexes as well as for the ligand-free 22GT fragment. All simulations were carried out using the standard force field AMBER 14 modified with the recently developed parmbsc0 parameters. The relevant structure was placed in the cell with such dimensions that the water molecules reached a density corresponding to the pressure of 1 bar. The cell was filled with K^+^ and Cl^–^ ions as described above. The pH was adjusted to 7.4, and the temperature to 298 K.

## 4. Conclusions

Taken together, we identified the thermodynamic forces responsible for topology changes of a human telomere fragment induced by ligand binding and temperature changes. Importantly, information on DNA conformational states that may be populated at different ligand and salt concentrations and temperatures, derived from mass and NMR spectroscopy analysis, was successfully incorporated into the model analysis of the calorimetric data. In this way, we obtained thermodynamic parameters that better describe the complex conformational phase space of G-quadruplexes.

## Figures and Tables

**Table 1:** Standard thermodynamic parameters of 22GT and 22AG folding (*T* = 25 °C, [K^+^] = 100 mM) and the corresponding no. of exchanged K^+^ ions (*n*).

	U→I		I→Q		U→Q	
	22GT	22AG ^a^	22GT	22AG^a^	22GT	22AG ^a^
$\Delta G^{}$/kcal mol^−1^	−4.7 ± 0.7	−3.7 ± 0.1	−1.0 ± 0.2	−1.4 ± 0.1	−5.7 ± 0.9	−5.1 ± 0.2
$\Delta H^{}$/kcal mol^−1^	−34 ± 4	−23.6 ± 0.6	−22 ± 5	−22.4 ± 0.1	−56 ± 9	−46.0 ± 1.2
$\Delta C_{p}$/kcal mol^−1^ K^−1^	−0.22 ± 0.1	−0.42 ± 0.02	−0.1 ± 0.1	−0.0 ± 0.2	−0.32± 0.2	−0.42 ± 0.2
$T\Delta S^{}$/kcal mol^−1^	−29 ± 4	−19.9 ± 0.6	−21 ± 5	−21.0 ± 0.1	−50 ± 9	−40.9 ± 0.7
n	−1.3 ± 0.1	−1.4 ± 0.2	−0.4 ± 0.2	−0.7 ± 0.2	−1.7 ± 0.3	−2.1 ± 0.4

^a^ ref. [40].

**Table 2:** Standard thermodynamic parameters of 360A binding to 22GT (*T* = 25 °C, [K^+^] = 100 mM) and the corresponding specific contributions to $\Delta G^{}$ estimated as shown in the text.

	Site 1	Site 2
$\Delta C_{P}^{}$/kcal mol^−1^ K^−1^	−0.02 ± 0.01	−0.16 ± 0.04
$\Delta H^{}$/kcal mol^−1^	−18 ± 4	−20 ± 5
$T\Delta S^{}$/kcal mol^−1^	−8 ± 4	−11 ± 5
$\Delta G^{}$/kcal mol^−1^	−9.6 ± 0.6	−8.7 ± 0.5
∆***G***_int_/kcal mol^−1^	−18 ± 4	−20 ± 5
∆***G***_hyd_/kcal mol^−1^	−1.6 ± 0.2	−12.8 ± 1.5
∆***G***_other_/kcal mol^−1^	10 ± 4	24 ± 5

## Data Availability

Data are contained within the article.
